# Synthetic RNAs for Gene Regulation: Design Principles and Computational Tools

**DOI:** 10.3389/fbioe.2014.00065

**Published:** 2014-12-11

**Authors:** Alessandro Laganà, Dennis Shasha, Carlo Maria Croce

**Affiliations:** ^1^Department of Molecular Virology, Immunology and Medical Genetics, Comprehensive Cancer Center, The Ohio State University, Columbus, OH, USA; ^2^Courant Institute of Mathematical Sciences, New York University, New York, NY, USA

**Keywords:** RNAi, siRNA, miRNA, a-miR, AntagomiR, Sponge, CRISPRi

## Abstract

The use of synthetic non-coding RNAs for post-transcriptional regulation of gene expression has not only become a standard laboratory tool for gene functional studies but it has also opened up new perspectives in the design of new and potentially promising therapeutic strategies. Bioinformatics has provided researchers with a variety of tools for the design, the analysis, and the evaluation of RNAi agents such as small-interfering RNA (siRNA), short-hairpin RNA (shRNA), artificial microRNA (a-miR), and microRNA sponges. More recently, a new system for genome engineering based on the bacterial CRISPR-Cas9 system (Clustered Regularly Interspaced Short Palindromic Repeats), was shown to have the potential to also regulate gene expression at both transcriptional and post-transcriptional level in a more specific way. In this mini review, we present RNAi and CRISPRi design principles and discuss the advantages and limitations of the current design approaches.

## Introduction

Natural regulatory RNAs are a heterogenous group of endogenous non-coding RNAs that modulate biological processes at many levels through different mechanisms. They have inspired the design of synthetic RNA molecules, such as riboswitches, sensors, and controllers, as key elements for programing cellular behaviors, as well as antisense-based approaches for specific gene expression regulation, which is the focus of this mini-review (Sharma et al., 2008; Culler et al., 2010; Liang et al., 2011).

RNA interference (RNAi) was discovered in 1998, when Andrew Fire and Craig C. Mello reported the capability of exogenous double-stranded RNAs (dsRNA) to silence genes in a specific manner in *C. elegans* (Fire et al., 1998). Central molecules in RNAi are microRNA (miRNA) and small-interfering RNA (siRNA).

miRNAs are small endogenous non-coding RNAs, typically 18–22 bp long, which derive from longer hairpin-shaped precursors called pre-miRNA (Bartel, 2004). A pre-miRNA can encode one or two different mature miRNAs, one from each arm (-5p and -3p). Pre-miRNAs come, in turn, from primary transcripts, called pri-miRNA, which are transcribed from miRNA genes. Mature miRNAs are incorporated into effector protein complexes called RISCs (RNA-induced silencing complex) and exert their regulatory function by binding specific target mRNAs through perfect or, more often, partial sequence complementarity, leading to the inhibition of their translation or promoting their degradation.

siRNAs are mostly exogenous dsRNA molecules derived from viral RNAs or artificially introduced into the cell (Chu and Rana, 2007).

The use of artificially designed siRNA has become a common and powerful strategy for the knock-down of gene expression yielding functional including therapeutic phenotypes (Gunsalus and Piano, 2005; Kim and Rossi, 2007). Several optimizations have been proposed in order to improve their efficacy and specificity (Liu et al., 2012b). Although research is focused on the development of selective delivery systems, a crucial factor is the presence of undesired off-target effects. siRNAs are designed to be perfectly complementary to their target sequences, ideally with few or no off-target genes. However, several studies have shown that a siRNA can bind mRNAs through partial complementarity, in a miRNA-like way, thus leading to undesirable and not easily predictable side effects (Birmingham et al., 2006; Jackson et al., 2006). In fact, despite the advances made in the recent past years, miRNA-target recognition has revealed itself to be a very dynamic mechanism influenced by many factors, which are only partially understood (Bartel, 2009; Thomas et al., 2010).

Along with specific gene silencing, the artificial repression of miRNAs can also provide a valuable tool for functional studies and have important therapeutic applications (Esquela-Kerscher and Slack, 2006; Garofalo et al., 2008; Croce, 2009). Two different strategies have been developed for the specific inhibition of miRNAs: antagomiRs and miRNA sponges (Krützfeldt et al., 2005; Ebert et al., 2007). The former consist of small RNAs exhibiting anti-complementarity to the miRNA to repress. The latter are longer RNA transcripts that act as attractors for miRNAs by distracting them from their original targets.

Finally, a novel methodology for artificial gene expression and miRNA regulation based on Clustered Regularly Interspaced Short Palindromic Repeats (CRISPR) has been recently proposed (Qi et al., 2013). CRISPR interference (CRISPRi) employs an engineered CRISPR/Cas system to control gene expression at the transcriptional level through a catalytically inactive Cas9 protein. Recent studies have shown that the CRISPR/Cas system can also target RNA (Hale et al., 2009).

In this mini-review, we summarize RNAi and CRISPRi design principles and discuss the advantages and limitations of the current approaches.

## siRNA Design Principles

siRNA are usually synthesized as double-stranded RNA duplexes or as hairpin-shaped molecules called shRNA. The siRNA design process consists of the identification of a functional binding site on a target mRNA sequence, which will correspond to the sense strand of the siRNA. The anti-sense sequence is obtained as the complement to the sense strand.

**Figure 1:**
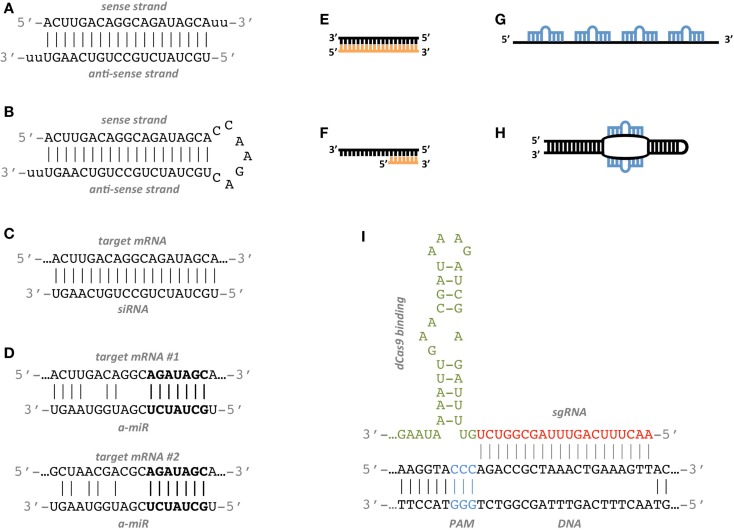
**Artificial RNA constructs for miRNA and gene regulation**. **(A)** Standard double strand siRNA; the anti-sense strand is the active agent which binds the target site. **(B)** shRNA construct; it is produced inside the target cell from a DNA construct that has been delivered to the nucleus and it expresses the anti-sense active strand. **(C)** The siRNA anti-sense strand binds the target mRNA with perfect complementarity. **(D)** Example of an a-miR sequence targeting two different sites with partial complementarity. The seed sequence of the a-miR, highlighted in bold characters, matches perfectly the target sites. **(E)** The antagomiR sequence (orange) perfectly matches the sequence of the target miRNA (black). **(F)** The Tiny LNA sequence (orange) perfectly matches the seed sequence of the target miRNA (black). **(G)** miRNA sponge construct with four miRNA binding sites separated by spacers. **(H)** Synthetic TUD construct with two exposed miRNA binding sites. **(I)** Model of a CRISPR sgRNA sequence binding the target DNA region. The PAM sequence (blue) is a short DNA motif juxtaposed to the DNA complementary region. The base-pairing nucleotides of the sgRNA are shown in red, while the dCas9-binding hairpin is in green.

Many studies have been conducted to determine the features associated to functional siRNAs and have allowed to establish siRNA design rules. Elbashir et al. (2001b) suggest to choose the 23-nt sequence motif AA(N19)TT as binding site, where N19 means any combination of 19 nucleotides (nt) and corresponds to the sense strand of the siRNA. The complement to AA(N19) corresponds to the anti-sense strand (Figures 1A–C).

Symmetric 3′ dTdT overhangs are added to the siRNA duplex to improve its stability and facilitate RISC loading. Although other combinations of nucleotides are acceptable, dGdG overhangs should be avoided, as they appear to be associated to decreased siRNA activity (Elbashir et al., 2001a,b; Strapps et al., 2010). siRNA duplexes often have asymmetric loading of the anti-sense versus sense strands. The strand whose 5′ end is thermodynamically less stable is preferentially incorporated into the RISC (Khvorova et al., 2003).

siRNA design rules can be classified into sequence and structure rules (See Table S1 in Supplementary Material). Sequence rules concern the position of the binding site in the target transcript and its nucleotide composition. The target region should be chosen preferably 50-100 nt downstream of the start codon and should avoid the middle of the coding sequence (Elbashir et al., 2001a; Hsieh et al., 2004). The G/C content of the binding site, and consequently of the siRNA, is relevant to the silencing activity and should be in the range of 30–55%, although values as low as 25% or as high as 79% are still associated to functional siRNAs (Reynolds et al., 2004; Liu et al., 2012a).

Numerous sequence rules regard the selection of nucleotides to prefer or avoid in specific positions of either the sense or the anti-sense strand of the duplex. For example, a higher content of A/U nucleotides in the 5′ end of the anti-sense strand of the siRNA yields higher silencing efficacy (Ui-Tei et al., 2004; Shabalina et al., 2006). Also, the 5′ half of the anti-sense strand dictates competition potency of siRNAs, which is a consequence of the RNAi machinery saturation followed by transfection of multiple siRNAs (Yoo et al., 2008).

Other relevant sequence features include the absence of internal repeats and the presence/absence of specific motifs (Reynolds et al., 2004).

Structure rules refer to the thermodynamics features of the siRNA/target duplex and are mostly expressed in terms of the nucleotide composition of the duplex itself or of the area surrounding the binding site (Chalk et al., 2004; Shabalina et al., 2006). Structure rules specify functional levels of binding energy at different positions of the duplex, and optimal energy difference between different positions of the duplex itself. Another important thermodynamic feature associated to siRNA efficacy is the structural accessibility of the target site. It has been demonstrated, in fact, that an mRNA stretch, which is not involved in a strict secondary structure exhibits a stronger binding affinity to a siRNA (or miRNA) molecule than one with a highly structured conformation (Tafer et al., 2008).

Several optimizations have been proposed to improve the activity of siRNA molecules, such as a more accurate prediction of the active strand of the duplex, design rules to avoid competition with endogenous miRNAs and vectors expressing multiple siRNAs at once (Cheng et al., 2009; Ma et al., 2014; Malefyt et al., 2014).

Many tools are available online for the design of siRNA and shRNA molecules (see Table 1).

**Table 1:** **Computational tools for siRNA, a-miR and CRISPR design**.

Tool	URL	Reference
**siRNA Design Tools**
OptiRNAi 2.0	http://rnai.nci.nih.gov	Cui et al. (2004)
siDirect 2	http://sidirect2.rnai.jp	Naito et al. (2009)
siRNA Scales	http://gesteland.genetics.utah.edu/siRNA_scales	Matveeva et al. (2007)
siExplorer	http://rna.chem.t.u-tokyo.ac.jp/cgi/siexplorer.htm	Katoh and Suzuki (2007)
RFRCDB-siRNA	http://www.bioinf.seu.edu.cn/siRNA/index.htm	Jiang et al. (2007)
OligoWalk	http://rna.urmc.rochester.edu/cgi-bin/server_exe/oligowalk/oligowalk_form.cgi	Lu and Mathews (2008)
Sfold	http://sfold.wadsworth.org	Ding et al. (2004)
siMAX	http://www.operon.com/products/siRNA/sirna-overview.aspx	Schramm and Ramey (2005)
DSIR	http://biodev.cea.fr/DSIR/	Vert et al. (2006)
siRNA Scan	http://bioinfo2.noble.org/RNAiScan.htm	Xu et al. (2006)
RNAxs	http://rna.tbi.univie.ac.at/cgi-bin/RNAxs	Tafer et al. (2008)
i-Score	http://www.med.nagoya-u.ac.jp/neurogenetics/i_Score/i_score.html	Ichihara et al. (2007)
siVirus	http://sivirus.rnai.jp	Naito et al. (2006)
**a-miR Design Tools**
miR-Synth	http://microrna.osumc.edu/mir-synth/	Lagana et al. (2014)
**CRISPR Design Tools**
Cas9 Design	http://cas9.cbi.pku.edu.cn	Ma et al. (2013)
CRISPR Design	http://crispr.mit.edu	Hsu et al. (2013)
Broad Inst. sgRNA Designer	http://www.broadinstitute.org/rnai/public/analysis-tools/sgrna-design	Doench et al. (2014)
sgRNAcas9	http://www.biootools.com	Xie et al. (2014)
CRISPR Genome Analyzer	http://crispr-ga.net	Guell et al. (2014)
CasOT	http://eendb.zfgenetics.org/casot	Xiao et al. (2014)
DNA 2.0 gRNA Design Tool	https://www.dna20.com/eCommerce/cas9/input	Cong et al. (2013); Ran et al. (2013)
E-CRISP	http://www.e-crisp.org/E-CRISP/	Heigwer et al. (2014)
ZiFiT	http://zifit.partners.org/ZiFiT/	Hwang et al. (2013)
CHOPCHOP	https://chopchop.rc.fas.harvard.edu	Montague et al. (2014)
CRISPRseek	http://www.bioconductor.org/packages/release/bioc/html/CRISPRseek.html	Zhu et al. (2014)
SSFinder	https://code.google.com/p/ssfinder/	Upadhyay and Sharma (2014)

*URLs and references are given for each tool*.

## Off-Targets, Multiple Targets, and the a-miR Approach

Although siRNAs and shRNAs are designed to specifically target a single gene through perfect complementarity to the binding site, several studies show that they can partially bind to many other transcripts in a way reminiscent of the endogenous miRNAs (Birmingham et al., 2006; Jackson et al., 2006). A single miRNA can potentially regulate hundreds of different mRNAs through partial sequence complementarity. In particular, perfect base pairing of the 5′ end region of the miRNA, termed “seed,” to a binding site located in the 3′ UTR of a mRNA, is usually sufficient to yield a significant repression of the target, while other recent studies also report functional centered site-mediated interactions (Shin et al., 2010; Helwak et al., 2013; Martin et al., 2014).

This represents a relevant drawback of single-target siRNAs, especially when pools of four or five siRNA duplexes per target gene are used to achieve stronger repression but also leading to widespread off-target effects.

One approach to the off-targeting problem consists of employing pools of siRNAs, at low concentrations, that target a single gene in multiple sites (Straka and Boese, 2010). The advantage of this approach lies in the fact that such pools are both effective on that one target, while the effects of a low concentrations siRNA on other potential targets should be negligible (Arvey et al., 2010; Larsson et al., 2010). Another study showed that siRNAs with a bulge at position 2 of the anti-sense strand were able to discriminate better between perfectly matched and mismatched targets (Dua et al., 2009; Li et al., 2010).

Targeting multiple genes can also be an intended choice, as there are many biological and biomedical applications in which it is important to regulate multiple genes at once while suffering as few side effects as possible. One way to achieve this goal is to exploit the multi-targeting properties of endogenous miRNAs by employing artificially designed miRNAs, or a-miRs. Two recent papers have shown that a-miRs can successfully repress at least two targets simultaneously by binding to one or more sites in their 3′ UTRs (Figure 1D) (Arroyo et al., 2014; Lagana et al., 2014). The employment of a single multi-target a-miR in place of a pair or a pool of single-target siRNAs is likely to yield significant repression of targets with few off-target effects.

## Silencing the Silencers: Antagomirs and miRNA Sponges

While the inhibition of over-expressed genes has been the main goal of RNAi research for years, the de-repression of down-regulated miRNA targets has increasingly gained importance over time. The inhibition of endogenous miRNAs was first introduced in 2005 by Krützfeldt et al. (2005). They employed cholesterol-conjugated oligo-ribonucleotides, which they termed “antagomiRs,” reproducing the anti-sense strand of the endogenous miRNA they inhibit. Their design is thus straightforward, as there is not much space for sequence variations (Figure 1E). Since then, a variety of chemical modifications have been proposed in order to increase binding affinity, improve nuclease resistance and facilitate *in vivo* delivery. They include locked nucleic acid (LNA), which possesses the highest affinity toward complementary RNA, Bifunctional oligodeoxynucleotide/antagomiR constructs, which ensure high transfection efficiency and prevention of unintended immune stimulation, and morpholino oligomers, which have been shown to be efficient inhibitors of both pri-miRNA and mature miRNA activity in zebrafish and *Xenopus laevis* (Summerton and Weller, 1997; Braasch and Corey, 2001; Petersen and Wengel, 2003; Ziegler et al., 2013). A further variant of antagomiRs is represented by short seed-targeting LNA oligonucleotides, called tiny LNAs. These molecules allow simultaneous inhibition of miRNAs within families sharing the same seed (Figure 1F) (Obad et al., 2011).

AntagomiRs represent one well-established tool for miRNA functional studies, and several works have also shown successful employment of antagomiRs as therapeutic agents able to restore disease-associated pathways altered by miRNA up-regulation. Like siRNAs, AntagomiRs can also have significant off-target effects, as they act like endogenous miRNAs and may hit complementary mRNA transcripts. However, experiments have showed no detectable effect on mRNAs with perfect tiny LNA complementary sites, not even at the proteomic level (Obad et al., 2011).

miRNA sponges are an alternative to antagomiRs. They act as competitive inhibitors that distract endogenous miRNAs from their natural targets. Many sponge variants have been described, such as miRNA-target mimics, miRNA decoys, and miRNA erasers, and they all consist of RNA constructs containing multiple binding sites for the miRNA to be sponged (Figure 1G) (Carè et al., 2007; Ebert et al., 2007; Franco-Zorrilla et al., 2007; Sayed et al., 2008).

A basic sponge consists of an RNA sequence exhibiting 4–10 miRNA binding sites separated by short spacers, usually 2–4 nt long. These sites can be either bulged or perfectly complementary to the miRNAs. In the first case, a bulge at positions 9–12 of the binding site is introduced in order to prevent cleavage and degradation of the sponge. Sponges with bulged binding sites produce stronger de-repressive effects than sponges with perfect binding sites (Ebert et al., 2007). Kluiver et al. (2012) developed a methodology for the rapid generation of miRNA sponges by making use of simple constructs with up to 20 perfect or bulged miRNA binding sites.

Structural optimizations have also been proposed. TuD RNAs (tough decoy RNAs) are efficient sponges with structurally accessible and indigestible miRNA binding sites (Figure 1H) (Haraguchi et al., 2009, 2012). The optimal TuD RNA consists of a bulged stem-loop structure where both sides of the bulge are miRNA binding sites which are perfectly complementary to the miRNA sequence and which do not form any base-pairing regions longer than 9nt.

## CRISPRi: The Gene Silencing Revolution

An exciting and promising advance in the field of artificial gene regulation comes from Clustered Regularly Interspaced Short Palindromic Repeats (CRISPR). CRISPR is a natural adaptive immune system used by archaea and bacteria against phage and plasmids (Jinek et al., 2012). This system is genomically encoded by the prokaryotic chromosome and consists of a series of short repeats separated by spacer sequences that match previously encountered foreign DNA. Thus, CRISPR arrays are transcribed and processed in order to produce mature crRNAs, which are loaded onto effector protein complexes and function as a guide to target recognition and degradation.

The CRISPR/Cas system has been engineered to function with synthetic small guide RNA (sgRNA) in order to perform genome editing in eukaryotes (Mali et al., 2013) (See Table S2 in Supplementary Material). The sgRNA consists of a 20 nt crRNA sequence complementary to the target region followed by a 42 nt Cas9-binding hairpin and a 40 nt transcription terminator. The target region must be of the form N20NGG that is any 21 nucleotides followed by GG. NGG is the 3′ protospacer-adjacent motif (PAM) and is required for Cas9 binding. This particular PAM sequence is derived from *Streptococcus pyogenes*, but other functional PAM sequences have been characterized from other bacteria (Esvelt et al., 2013). In addition, if a U6 snRNA or T7 promoters are used to express the sgRNA, this must start with G or GG, respectively, in order to maintain transcript initiation. Thus, the target region must be of the form GN19NGG or GGN18NGG. Ultimately, the beginning of the sgRNA and the PAM sequence will depend on the specific promoters and Cas9 used (Figure 1I).

DNA breaks caused by Cas9 are repaired through either homologous recombination or non-homologous end joining (NHEJ) mechanisms, thus this system can be used to either disrupt or edit a gene (i.e., insertions and deletions). Many tools are currently available online for the design of sgRNAs (Table 1).

Besides genome-editing applications, the CRISPR/Cas9 system can be employed for gene expression regulation. The system, known as CRISPR interference (CRISPRi), is based on a catalytically dead Cas9 (dCas9) lacking endonuclease activity co-expressed with a sgRNA (Gilbert et al., 2013; Qi et al., 2013). Instead of generating DNA breaks, the recognition complex interferes with transcriptional elongation, RNA polymerase binding, or transcription factor binding, leading to efficient inhibition of gene expression. CRISPRi gene silencing is inducible and reversible and recognition of the targets depends solely on the sgRNA sequence (Qi et al., 2013).

A “seed” region has been identified as the 12nt region adjacent to the PAM site. Mismatches in the seed region can dramatically reduce the repression, while mismatches in the non-seed area can cause a mild effect. Design guidelines recommend using a length of 20–25 nt as the base-pairing region of the sgRNA (Larson et al., 2013) and provide specific design rules based on nucleotide preference for active sgRNA (Doench et al., 2014). A recent study aimed at the identification of features of effective sgRNA specific to CRISPRi, suggests that the target site should be chosen from −50 to +300 bp relative to the Transcription Start Site (TSS) of a gene (Gilbert et al., 2014). The authors observed that nucleotide homopolymers have a strongly negative effect on sgRNA activity and that the GC content of the sgRNA or the binding site is not correlated with sgRNA activity, although another study reports a decreased activity of sgRNA with low or high GC content (Doench et al., 2014). Moreover, CRISPRi activity seems to be highly sensitive to mismatches between the sgRNA and DNA sequence, thus the authors conclude that properly designed sgRNA will have minimal off-target effects. However, previous studies reported silencing activity with sgRNAs exhibiting mismatches to the target in the seed area (Cradick et al., 2013) and that off-targets might be cell type dependent and determined by various complicated factors in addition to primary DNA sequences (Duan et al., 2014). Thus, side effects still constitute a challenge, which needs to be properly addressed by further focused research.

Gilbert et al. also introduced the sunCas9 CRISPRa system, in which expression of a single sgRNA with one binding site is sufficient to turn on genes that are poorly expressed or that increase the expression of well-expressed genes (Gilbert et al., 2014; Tanenbaum et al., 2014).

CRISPRi can also be successfully employed to knock out miRNAs, by using a sgRNA/Cas9 complex targeting the pre-miRNA sequence (Zhao et al., 2014), and to study functional miRNA-target interactions *in vivo* by site-specific genome engineering (Bassett et al., 2014).

Finally, although current tools for CRISPRi are based on the DNA targeting approach described above, the discovery of other Cas proteins targeting RNA molecules, such as Cmr, suggests an alternative post-transcriptional methodology similar to RNAi (Hale et al., 2009; Zebec et al., 2014).

## Conclusion

Both RNAi and CRISPRi represent valid approaches for artificial gene regulation and both can suffer from significant side effects which may result from factors beyond sequence match. One clear advantage of CRISPRi over RNAi is that being an exogenous system it does not compete with the endogenous machinery of miRNA processing. Nevertheless, both techniques require more work in terms of enhancing targeting efficiency and reducing side effects.

## Conflict of Interest Statement

The authors declare that the research was conducted in the absence of any commercial or financial relationships that could be construed as a potential conflict of interest.

## Supplementary Material

The Supplementary Material for this article can be found online at http://www.frontiersin.org/Journal/10.3389/fbioe.2014.00065/abstract
