# Supplementary material for: Synthetic RNAs for Gene Regulation: Design Principles and Computational Tools
Source: Front Bioeng Biotechnol. 2014 Dec 11;2:65. doi: 10.3389/fbioe.2014.00065 (PMC4263176; doi:10.3389/fbioe.2014.00065)
Supplement: Supplementary file 1 [file Data_Sheet_1.PDF]

Alessandro Laganà[1,*], Dennis Shasha[2], Carlo M. Croce[1]

Synthetic RNAs for gene regulation: design principles and computational tools

[1]Department of Molecular Virology, Immunology and Medical Genetics, Comprehensive Cancer Center, The Ohio State University, Columbus, OH, USA
[2]Courant Institute of Mathematical Sciences, New York University, New York, NY, USA

*** Correspondence:** Alessandro Laganà, Department of Molecular Virology, Immunology and Medical Genetics, Comprehensive Cancer Center, The Ohio State University, 460 W 12th Avenue, Room 0995, Columbus, OH 43210, USA. E-mail: alessandro.lagana@osumc.edu.

SUPPLEMENTARY TABLES

Table S1. siRNA design rules. References are given for each rule.

Design Rule	Reference
**Sequence Rules**	
Select the target region preferably 50-100 nt downstream of the start codon	(Elbashir et al., 2001)
Avoid to target the middle of the coding sequence of target gene	(Hsieh et al., 2004)
Pooling of four or five siRNA duplexes per gene	(Hsieh et al., 2004)
Antisense strand with higher information content	(Peek, 2007)
5' half of the antisense strand dictates competition potency of siRNAs	(Yoo et al., 2008)
Bulge at position 2 of the antisense reduces off-targets	(Dua et al., 2009; Li et al., 2010)
Absence of any GC stretch >9 nt long	(Ui-Tei et al., 2004)
At least five A/U residues in the 5' terminal one-third of the antisense strand	(Ui-Tei et al., 2004; Shabalina et al., 2006; Vert et al., 2006)
A higher 'A/U' content in the 3' end than that in the 5' end (sense strand)	(Amarzguioui and Prydz, 2004a)
G/C content ranges: 32-58%, 30-52%, 32-79%, 36-53%, 35-73%, 25-55%	(Amarzguioui and Prydz, 2004b; Reynolds et al., 2004; Elbashir et al., 2001; Chalk et al., 2004; Klingelhoefer et al., 2009; Liu et al., 2012)
Absence of internal repeats	(Reynolds et al., 2004)
Presence of motifs 'AAC', 'UC', 'UG', 'AAG', 'AGC', 'UCU', 'UCCG', 'CUU', 'CU', 'GUU', 'UCC', 'CG', 'AUC', 'GCG', 'UUU', 'ACA', 'UUC', 'CAA' in antisense strand	(Vert et al., 2006; Klingelhoefer et al., 2009; Liu et al., 2012; Wang et al., 2010)
Avoid motifs 'CUU', 'CUA', 'GUU', 'GU', 'GAU', 'ACGA', 'GCC', 'GUGG', 'CCC', 'GGC', 'CCG', 'GGG', 'CAG', 'GAG', 'GCA', 'AUA', 'CUG', 'AG', 'GG', 'GGA' in antisense strand	(Vert et al., 2006; Klingelhoefer et al., 2009; Liu et al., 2012; Wang et al., 2010)
High content of 'U' in antisense strand	(Wang et al., 2010)
Low content of 'G' in antisense strand	(Wang et al., 2010)
**Structure Rules**	
Total hairpin energy < 1	(Chalk et al., 2004)
Antisense 5' end binding energy < 9	(Chalk et al., 2004)
Sense 5' end binding energy in range 5-9	(Chalk et al., 2004)
Middle binding energy < 13	(Chalk et al., 2004)
Energy difference < 0	(Chalk et al., 2004)
Energy difference within -1 and 0	(Chalk et al., 2004)
Significant ΔG difference between positions 1 and 18	(Shabalina et al., 2006)
High ΔG in positions 1-4, 5-8 and 13-14 in the antisense strand	(Klingelhoefer et al., 2009)
Low ΔG in positions 18-19 in the antisense strand	(Klingelhoefer et al., 2009)
Avoid folding of siRNA	(Klingelhoefer et al., 2009)

Table S2. CRISPR sgRNA design rules. References are given for each rule.

Design Rule	Reference
Standard target region form: N20NGG (any 21 nucleotides followed by GG)	(Mali et al., 2013)
Target region form when using a U6 snRNA promoter: GN19NGG	(Mali et al., 2013)
Target region form when using a T7 promoter: GGN18NGG	(Mali et al., 2013)
Seed region: 12nt region adjacent to the PAM site	(Larson et al., 2013)
Length of the base-pairing region of the sgRNA: 20-25 nt	(Larson et al., 2013)
Position of target site for CRISPRi: -50 to +300 bp relative to the TSS of a gene	(Gilbert et al., 2014)
Nucleotide homopolymers have a strongly negative effect on sgRNA activity	(Gilbert et al., 2014)
GC content of the sgRNA or the binding site is not correlated with sgRNA activity	(Gilbert et al., 2014)
Decreased activity of sgRNA with low or high GC content	(Doench et al., 2014)
CRISPRi activity is highly sensitive to mismatches between the sgRNA and DNA sequence	(Gilbert et al., 2014)
Mismatches between the sgRNA and DNA sequence might be tolerated / Off-targets might be cell-type dependent	(Cradick et al., 2013; Duan et al., 2014)
Position of target site for CRISPRa: -400 to -50 bp upstream from the TSS	(Tanenbaum et al., 2014)

REFERENCES

Amarzguioui, M., and Prydz, H. (2004a). An algorithm for selection of functional siRNA sequences. *Biochem Biophys Res Commun* 316, 1050–1058. doi:10.1016/j.bbrc.2004.02.157.

Amarzguioui, M., and Prydz, H. (2004b). An algorithm for selection of functional siRNA sequences. *Biochem Biophys Res Commun* 316, 1050–1058. doi:10.1016/j.bbrc.2004.02.157.

Chalk, A. M., Wahlestedt, C., and Sonnhammer, E. L. L. (2004). Improved and automated prediction of effective siRNA. *Biochem Biophys Res Commun* 319, 264–274. doi:10.1016/j.bbrc.2004.04.181.

Cradick, T. J., Fine, E. J., Antico, C. J., and Bao, G. (2013). CRISPR/Cas9 systems targeting -globin and CCR5 genes have substantial off-target activity. *Nucleic Acids Res* 41, 9584–9592. doi:10.1093/nar/gkt714.

Doench, J. G., Hartenian, E., Graham, D. B., Tothova, Z., Hegde, M., Smith, I., Sullender, M., Ebert, B. L., Xavier, R. J., and Root, D. E. (2014). Rational design of highly active sgRNAs for CRISPR-Cas9–mediated gene inactivation. *Nat Biotechnol*, 1–8. doi:10.1038/nbt.3026.

Dua, P., Yoo, J. W., Kim, S., and Lee, D.-K. (2009). Modified siRNA Structure With a Single Nucleotide Bulge Overcomes Conventional siRNA-mediated Off-target Silencing. *Mol Ther* 19, 1676–1687. doi:10.1038/mt.2011.109.

Duan, J., Lu, G., Xie, Z., Lou, M., Luo, J., Guo, L., and Zhang, Y. (2014). Genome-wide identification of CRISPR/Cas9 off-targets in human genome. *Cell Research*, 1–4. doi:10.1038/cr.2014.87.

Elbashir, S. M., Lendeckel, W., and Tuschl, T. (2001). RNA interference is mediated by 21- and 22-nucleotide RNAs. *Genes Dev* 15, 188–200.

Gilbert, L. A., Horlbeck, M. A., Adamson, B., Villalta, J. E., Chen, Y., Whitehead, E. H., Guimaraes, C., Panning, B., Ploegh, H. L., Bassik, M. C., et al. (2014). Genome-Scale CRISPR-Mediated Control of

Gene Repression and Activation. *Cell* 159, 647–661. doi:10.1016/j.cell.2014.09.029.

Hsieh, A., Bo, R., Manola, J., Vazquez, F., Bare, O., Khvorova, A., Scaringe, S., and Sellers, W. (2004). A library of siRNA duplexes targeting the phosphoinositide 3-kinase pathway: determinants of gene silencing for use in cell-based screens.

Klingelhoefer, J. W., Moutsianas, L., and Holmes, C. (2009). Approximate Bayesian feature selection on a large meta-dataset offers novel insights on factors that effect siRNA potency. *Bioinformatics* 25, 1594–1601. doi:10.1093/bioinformatics/btp284.

Larson, M. H., Gilbert, L. A., Wang, X., Lim, W. A., Weissman, J. S., and Qi, L. S. (2013). CRISPR interference (CRISPRi) for sequence-specific control of gene expression. *Nature Protocols* 8, 2180–2196. doi:10.1038/nprot.2013.132.

Li, X., Yoo, J. W., Lee, J. H., Hahn, Y., Kim, S., and Lee, D.-K. (2010). Identification of sequence features that predict competition potency of siRNAs. *Biochem Biophys Res Commun* 398, 92–97. doi:10.1016/j.bbrc.2010.06.041.

Liu, Q., Zhou, H., Cui, J., Cao, Z., and Xu, Y. (2012). Reconsideration of In-Silico siRNA Design Based on Feature Selection: A Cross-Platform Data Integration Perspective. *PLoS ONE* 7, e37879. doi:10.1371/journal.pone.0037879.s016.

Mali, P., Yang, L., Esvelt, K. M., Aach, J., Guell, M., DiCarlo, J. E., Norville, J. E., and Church, G. M. (2013). RNA-Guided Human Genome Engineering via Cas9. *Science* 339, 823–826. doi:10.1126/science.1232033.

Peek, A. S. (2007). Improving model predictions for RNA interference activities that use support vector machine regression by combining and filtering features. *BMC Bioinformatics* 8, 182. doi:10.1186/1471-2105-8-182.

Reynolds, A., Leake, D., Boese, Q., Scaringe, S., Marshall, W. S., and Khvorova, A. (2004). Rational siRNA design for RNA interference. *Nat Biotechnol* 22, 326–330. doi:10.1038/nbt936.

Shabalina, S. A., Spiridonov, A. N., and Ogurtsov, A. Y. (2006). Computational models with thermodynamic and composition features improve siRNA design. *BMC Bioinformatics* 7, 65. doi:10.1186/1471-2105-7-65.

Tanenbaum, M. E., Gilbert, L. A., Qi, L. S., Weissman, J. S., and Vale, R. D. (2014). A Protein-Tagging System for Signal Amplification in Gene Expression and Fluorescence Imaging. *Cell* 159, 635–646. doi:10.1016/j.cell.2014.09.039.

Ui-Tei, K., Naito, Y., Takahashi, F., Haraguchi, T., Ohki-Hamazaki, H., Juni, A., Ueda, R., and Saigo, K. (2004). Guidelines for the selection of highly effective siRNA sequences for mammalian and chick RNA interference. *Nucleic Acids Res* 32, 936–948. doi:10.1093/nar/gkh247.

Vert, J. P., Foveau, N., Lajaunie, C., and Vandenbrouck, Y. (2006). An accurate and interpretable model for siRNA efficacy prediction. *BMC Bioinformatics* 7, 520. doi:10.1186/1471-2105-7-520.

Wang, L., Huang, C., and Yang, J. Y. (2010). Predicting siRNA potency with random forests and support vector machines. *BMC Genomics* 11, S2. doi:10.1186/1471-2164-11-S3-S2.

Yoo, J. W., Kim, S., and Lee, D.-K. (2008). Competition potency of siRNA is specified by the 5′-half sequence of the guide strand. *Biochem Biophys Res Commun* 367, 78–83. doi:10.1016/j.bbrc.2007.12.099.
